# Construction and integrated analysis of the ceRNA network hsa_circ_0000672/miR-516a-5p/TRAF6 and its potential function in atrial fibrillation

**DOI:** 10.1038/s41598-023-34851-z

**Published:** 2023-05-11

**Authors:** Xing Liu, Mingxing Wu, Yan He, Chun Gui, Weiming Wen, Zhiyuan Jiang, Guoqiang Zhong

**Affiliations:** 1 Department of Cardiology, Xiangtan Central Hospital, Xiangtan, China; 2 Department of Cardiology, The First Affiliated Hospital of Guangxi Medical University, Nanning, China

**Keywords:** Biomarkers, Cardiology, Arrhythmias

## Abstract

Atrial fibrosis is a crucial contributor to initiation and perpetuation of atrial fibrillation (AF). This study aimed to identify a circRNA-miRNA-mRNA competitive endogenous RNA (ceRNA) regulatory network related to atrial fibrosis in AF, especially to validate hsa_circ_0000672/hsa_miR-516a-5p/TRAF6 ceRNA axis in AF preliminarily. The circRNA-miRNA-mRNA ceRNA network associated with AF fibrosis was constructed using bioinformatic tools and literature reviews. Left atrium (LA) low voltage was used to represent LA fibrosis by using LA voltage matrix mapping. Ten controls with sinus rhythm (SR), and 20 patients with persistent AF including 12 patients with LA low voltage and 8 patients with LA normal voltage were enrolled in this study. The ceRNA regulatory network associated with atrial fibrosis was successfully constructed, which included up-regulated hsa_circ_0000672 and hsa_circ_0003916, down-regulated miR-516a-5p and five up-regulated hub genes (KRAS, SMAD2, TRAF6, MAPK11 and SMURF1). In addition, according to the results of Kyoto Encyclopedia of Genes and Genomes (KEGG) pathway analysis, these hub genes were clustered in TGF-beta and MAPK signaling pathway. In the patients with persistent AF, hsa_circ_0000672 expression in peripheral blood monocytes was significantly higher than those in controls with SR by quantitative real-time polymerase chain reaction (p-value < 0.001). Furthermore, hsa_circ_0000672 expression was higher in peripheral blood monocytes of persistent AF patients with LA low voltage than those with LA normal voltage (p-value = 0.002). The dual-luciferase activity assay confirmed that hsa_circ_0000672 exerted biological functions as a sponge of miR-516a-5p to regulate expression of its target gene TRAF6. Hsa_circ_0000672 expression in peripheral blood monocytes may be associated with atrial fibrosis. The hsa_circ_0000672 may be involved in atrial fibrosis by indirectly regulating TRAF6 as a ceRNA by sponging miR-516a-5p.

## Introduction

Atrial fibrillation (AF), which is one of the most prevalent cardiac arrhythmias, is becoming increasingly incident in aged population^1^. In addition, patients with AF had a significantly increased risk of heart failure, embolic stroke, cognitive impairment and mortality^1,2^. At present, the pathogenesis of AF is primarily driven by genes, inflammation, and atrial electrical and structural remodeling^3,4^. Atrial fibrosis is the most common characteristic change in structural remodeling, which is closely associated with the occurrence and maintenance of AF^3^. Furthermore, pre-existing atrial fibrosis increases the risk of AF recurrence after catheter ablation^5,6^. Currently, left atrium (LA) fibrosis can be clinically evaluated by late gadolinium enhancement cardiac magnetic resonance imaging (LGE-MRI) or LA low voltage using high-density bipolar LA voltage mapping^7^. However, these tests are costly or invasive, which limits their use as a routine method to assess left atrial fibrosis in AF in clinical practice. Therefore, it is crucial to fully understand the molecular mechanisms of atrial structural remodeling, and to find a more simple and non-invasive way to evaluate LA fibrosis in AF patients in clinical practice, which will improve the treatment effect and long-term prognosis of AF patients.

Circular RNAs (circRNAs) are closed circular single-stranded RNA molecules that have good specificity and stability^8^. As cells secrete circRNAs into the blood through exosomes, circRNAs level in blood may usually exceed those in other tissues^9^. Previous studies have shown that circRNAs have a promising application prospect as a potential biomarker in the cardiovascular diseases^10,11^. In addition, circRNAs can regulate the expression of protein-coding gene by sponging microRNA (miRNA or miR) in the circRNA-miRNA-mRNA competitive endogenous RNA (ceRNA) networks^8^. By specifically acting as a sponge of miRs, circRNAs are widely involved in the pathogenesis of cardiovascular diseases^12,13^. However, the circRNA-miRNA-mRNA ceRNA networks are rarely studied in atrial fibrosis.

**Figure 1:**
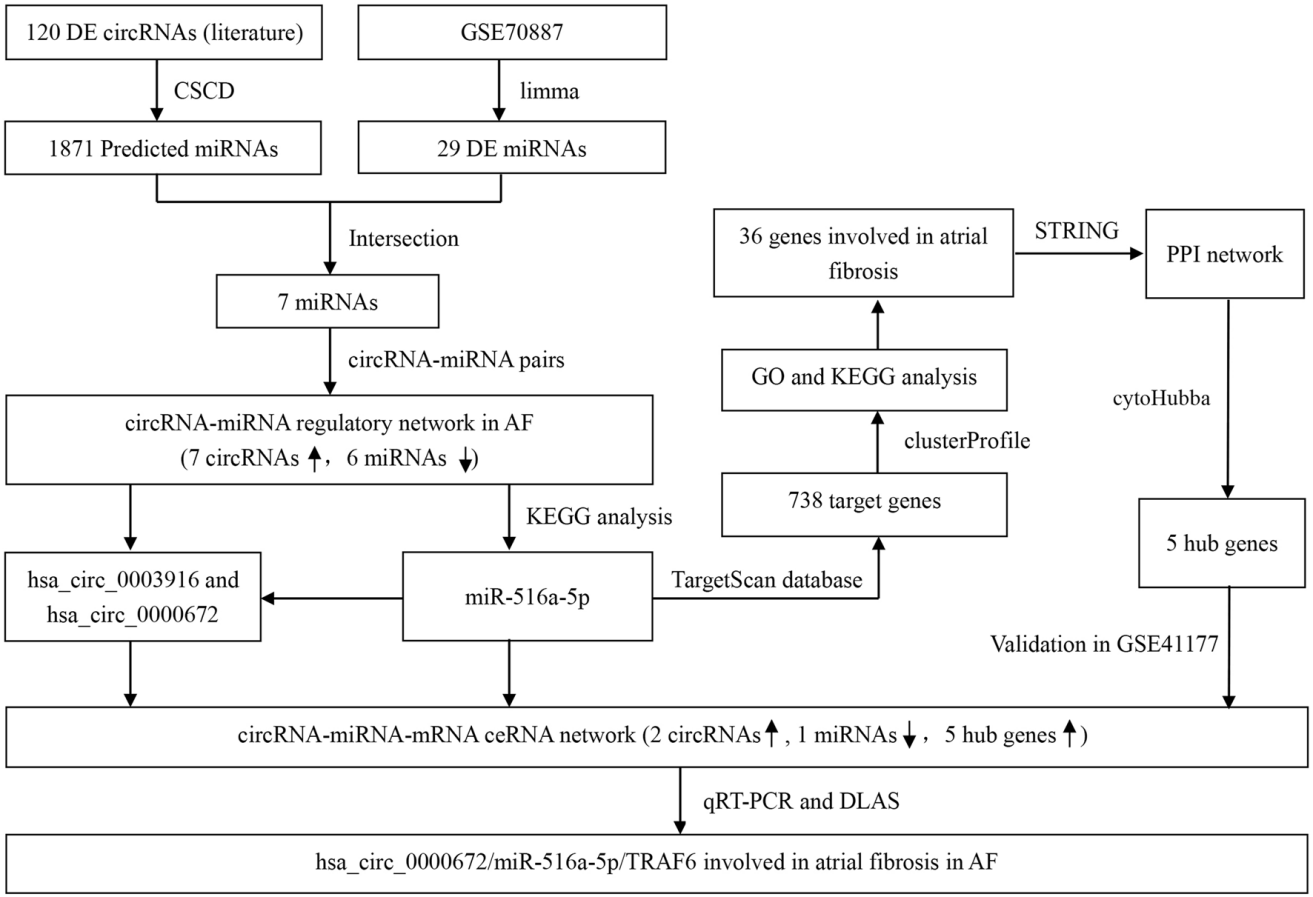
The flowchart of our study. ceRNA, competitive endogenous RNA; CSCD, cancer specific circRNA database; DE, diferentially expressed; DLAS, dual-luciferase activity assay; GO, gene ontology; KEGG, Kyoto Encyclopedia of Genes and Genomes; qRT-PCR, quantitative real-time polymerase chain reaction.

In this study, we initially screened the circRNA-miRNA-mRNA ceRNA networks associated with atrial fibrosis by reviewing literature and using bioinformatics tools. After that, quantitative real-time polymerase chain reaction (qRT-PCR) was used to validate the bioinformatically predicted circRNAs in patients with or without AF, and the circRNAs was further quantified between AF patients with LA low-voltage and LA normal-voltage. Finally, the specific binding of circRNA to miRNA and miRNA to mRNA were testified by double luciferase assay to preliminarily clarify the potential mechanism of circRNAs certified by qRT-PCR contributing to atrial fibrosis in vitro. The flowchart of our study is shown in Fig. 1.

## Materials and methods

### Data acquisition and identification of differentially expressed (DE) miRNAs and DE circRNAs in AF

One miRNA microarray dataset (GSE70887) related to AF was downloaded from gene expression omnibus (GEO, http://www.ncbi.nlm.nih.gov/geo/)^14^. Atrial appendage tissue from four patients with AF and two controls with sinus rhythm (SR) was included in the miRNA dataset GSE70887, and the samples were tested by the GPL19546 Agilent-021827 Human miRNA Microarray [miRBase release 17.0 miRNA ID version] platform. The Limma package for R software^15^ was utilized to screen differentially expressed (DE) miRNAs between AF and SR samples, using the following significant difference of the cut-off level that was |log2(fold change)|> 1 and p-value < 0.05. For visualization, volcano map and heat map of DE miRNAs were generated using the “pheatmap” and “ggplot2” packages in the R software. At present, there is no circRNA expression dataset from peripheral blood that compares the difference between AF and SR in GEO database. The published differential expression profile data of circRNAs in peripheral blood monocytes from 4 healthy participants and 4 AF patients by microarray was included in this bioinformatics study, to look for the circRNAs that may be utilized as indicators for diagnosis of atrial fibrosis^16^. A cut-off point that was |log2(fold change)|> 1 and p-value < 0.05 was used to detect DE circRNAs between AF and SR in their study^16^.

### Construction of circRNA-miRNA regulatory network

DE miRNAs and DE circRNAs were selected to construct the circRNA-miRNA network. According to the previously proposed ceRNA hypothesis^17^, the expression of circRNAs in the circRNA-miRNA network and their corresponding miRNAs showed an opposite trend. CircBase (http://www.circb ase.org/)^18^ was used to acquire information about circRNAs. The cancer specific circRNA database (CSCD, https://gb.whu.edu.cn/CSCD/)^19^ was utilized to obtain all predicted miRNAs for each DE circRNAs. Afterward, miRNAs that overlapped with DE miRNAs and predicted miRNAs were collected. Finally, based on these circRNA–miRNA pairs, the circRNA–miRNA network involved in AF was established and visualized using Cytoscape software (version 3.8.0). Boxplots of the expression level of these miRNAs in the circRNA–miRNA network in the GSE70887 dataset were generated using the “reshape2” and “ggpubr” packages in R software.

### GO and KEGG functional enrichment analysis

TargetScan database (http://www.targetscan.org/vert_72/)^20^ was utilized to predict targeted mRNAs of each miRNAs in the circRNA-miRNA network constructed in this study. Additionally, GO and KEGG functional enrichment analysis was performed for targeted mRNAs predicted by each miRNAs in the circRNA-miRNA network by using the “Clusterprofiler” package in R software^21–24^. A p-value < 0.05 was considered as statistically significant, and results were visualized using bubble charts. If KEGG enrichment results of target genes predicted by a miRNA showed more than 2 signaling pathways related to AF fibrosis, the miRNA and its potential target genes would be used for subsequent analysis. Otherwise, the miRNA would be removed from our analysis.

### Construction of protein–protein interaction (PPI) regulatory network and screening of hub genes

The potential target genes enriched in AF fibrosis-related signaling pathways screened by the above conditions were used to construct PPI networks. CytoCope 3.8.0 software was used to visualize the PPI network constructed with the STRING database (https://stringdb.org/)^25^. Then, the Degree algorithm in Cytoscape plug-in cytoHubba was used to calculate the degree of each protein node and screen the hub genes in PPI network.

### Validation of the trend of hub genes expression in AF

We downloaded an AF-related mRNA microarray dataset (GSE41177) from GEO database to validate the trend of hub genes expression in AF. The GSE41177 dataset included 32 atrial tissue samples with persistent AF and 6 atrial tissue samples with SR, and these samples were tested by the GPL570 Affymetrix Human Genome U133 Plus 2.0 platform. |log2(fold change)|> 0.5 and an false discovery rate (FDR) < 0.05 was used as the cut-off point of differential expression of hub genes in the GSE41177 dataset.

### Study population and collection of blood samples

According to the arrhythmia related radiofrequency ablation treatment guidelines^26,27^, twenty patients with persistent AF (PsAF group) and ten patients without AF with left accessory pathway-induced atrioventricular reentrant tachycardia (SR group) were enrolled in this study. AF that failed to self-terminate or requires cardioversion after more than seven days was defined as persistent AF^26^. All patients enrolled in this study underwent radiofrequency ablation after atrial septal puncture. All patients underwent LA voltage mapping. About 10 ml of peripheral blood was collected from each participant, monocytes were purified from peripheral blood using Human Peripheral Blood Monocyte Isolation Kit (Solarblo, China) and frozen for analysis. The project was approved by the Ethics Committee of the First Affiliated Hospital of Guangxi Medical University and followed the Helsinki Declaration's ethical principles. All patients signed informed consent forms. The study was registered at Chinese Clinical Trial Registry (http://www.chictr.org.cn, No.ChiCTR2200066444, Registered on 06/12/2022, Retrospectively registered).

### LA voltage mapping

A 20-pole multielectrode catheter (PentaRay Nav Catheter; Biosense Webster, USA) and an irrigated RF catheter (Thermocool® SmartTouch®; Biosense Webster, USA) was used to perform LA voltage mapping in the PsAF group and the SR group, respectively. Low-voltage zones (LVZs) under AF were defined as bipolar voltage < 0.5 mV^28–30^, while LVZs under sinus rhythm were considered as the sites displaying < 1.0 mV peak-to-peak bipolar voltage^30,31^. The LA body area minus the LA appendage, the pulmonary vein antrum regions, and the mitral valve were referred to as the LA surface area. The mean proportion of LVZs on the LA surface area is known as the low voltage area (LVA). Additionally, the atrial surface area and LVA were quantified by using standardized software (CARTO 3, Biosense Webster, USA). Patients with PsAF were divided into LVZs subgroup (n = 12) and non-LVZs subgroup (n = 8) according to the presence or absence of left atrial LVZs.

### Quantitative real-time polymerase chain reaction

Total RNA in monocytes was isolated with the TRIzol reagent (Invitrogen, Carlsbad, Calif., USA) following the manufacturer’s protocol. The reverse transcription was carried out using PrimeScript™ RT Master Mix (Takara, Tokyo, Japan). The RNA relative expression was evaluated by 2× SYBR Green Master Mix kit (Applied Biosystems, Carlsbad, CA) with special RT-qPCR primer sets in ABI 7500 platform (Applied Biosystems, Carlsbad, CA, USA). The cycle threshold (Ct) value of each gene was utilized to calculate relative expression of hsa_circ_0003916 and hsa_circ_0000672 using the 2^−ΔΔCt^ method. Glyceraldehyde-3-phosphate dehydrogenase (GAPDH) was used as the internal control for normalizing genes expression. The specific primers were listed in Table 1.

**Table 1:** Primer sequences.

Primer	Sequence
hsa_circ _0003916-F	5′- GAGGTTACGAGCAAAGGGAAT -3′
hsa_circ _0003916-R	5′- ATTTGCTGCACTTGTTGTGG -3′
hsa_circ_0000672-F	5′- GGGAGCCTGAGACACAGTTG -3′
hsa_circ _0000672-R	5′- CTTTTCTCCTCGTCCGTGGT -3′
GAPDH-F	5′-GTCTCCTCTGACTTCAACAGCG-3′
GAPDH-R	5′-ACCACCCTGTTGCTGTAGCCAA-3′

### Dual luciferase assay

The fluorescent luciferase reporter plasmids containing TRAF6-3′-UTR wild-type (wt), TRAF6-3′-UTR mutant-type (mut), hsa_circ_0000672 wt, hsa_circ_0000672 mut, hsa_circ_0003916 wt, or hsa_circ_0003916 mut, and hsa-miR-516a-5p mimic or negative control mimic were obtained from Hanheng Biological Technology (Shanghai, China). Following plating in 96-well plates, the HEK 293T cells were co-transfected with fluorescent luciferase reporter plasmids containing hsa_circ_0003916 wt or hsa_circ_0003916 mut and hsa-miR-516a-5p mimic or negative control mimic by using Lipofetamine 2000 (Invitrogen, Carlsbad, Calif., USA). Similarly, fluorescent luciferase reporter plasmids containing hsa_circ_0000672 wt or hsa_circ_0000672 mut and hsa-miR-516a-5p mimic or negative control mimic were co-transfected into the HEK 293T cells. The luciferase activity were determined with dual-luciferase Reporter Assay System (Promega, Madison, WI, USA) 48 h after transfection, and Firefly luciferase activity was normalized to that of Renilla. The same method was used to verify direct binding of hsa-miR-516a-5p to the 3′-UTR of TRAF6.

To further clarify that competitive relationship between hsa_circ_0000672 and TRAF6 for hsa-miR-516a-5p binding. The HEK 293T cells were co-transfected with fluorescent luciferase reporter plasmids containing TRAF6-3′-UTR wt or TRAF6-3′-UTR mut, and plasmids containing has_circ_0000672 wt, hsa_circ_0000672 mut, or blank vector, and hsa-miR-516a-5p mimic or negative control mimic, and the luciferase activity were measured by dual-luciferase Reporter Assay System (Promega, Madison, WI, USA) 48 h after transfection.

### Statistical analysis

The bioinformatics statistical analyses were performed by using packages mentioned above in R software (version 4.0.4). The empirical Bayes statistics in the “limma” package were used to compute moderated t-statistic and identify DE-miRNAs and DE-mRNAs for the GEO datasets. In each experiment, all measurements were performed in triplicate. The continuous variables were presented as means ± standard deviations. The categorical variables were presented as percentage. The two unpaired Student’s t-test was used to compare statistical significance of continuous variables between PsAF group and SR group, or between LVZs subgroup and non-LVZs subgroup. A Chi-squared test or Fisher’s exact test was used to compare statistical significance of categorical variables between PsAF group and SR group, or between LVZs subgroup and non-LVZs subgroup. A p-value < 0.05 was considered statistically significant. These analyses were performed using IBM SPSS version 26.0 (SPSS Inc., Chicago, Ill., USA) and GraphPad Prism 8 (GraphPad Software, San Diego, USA).

### Ethics approval and consent to participate

All experiments were approved by the Ethics Committee of the First Affiliated Hospital of Guangxi Medical University. All research was performed following relevant regulations, and informed consent was obtained from all participants before the sample collection.

## Results

### Identification of DE miRNAs and DE circRNAs in AF

**Figure 2:**
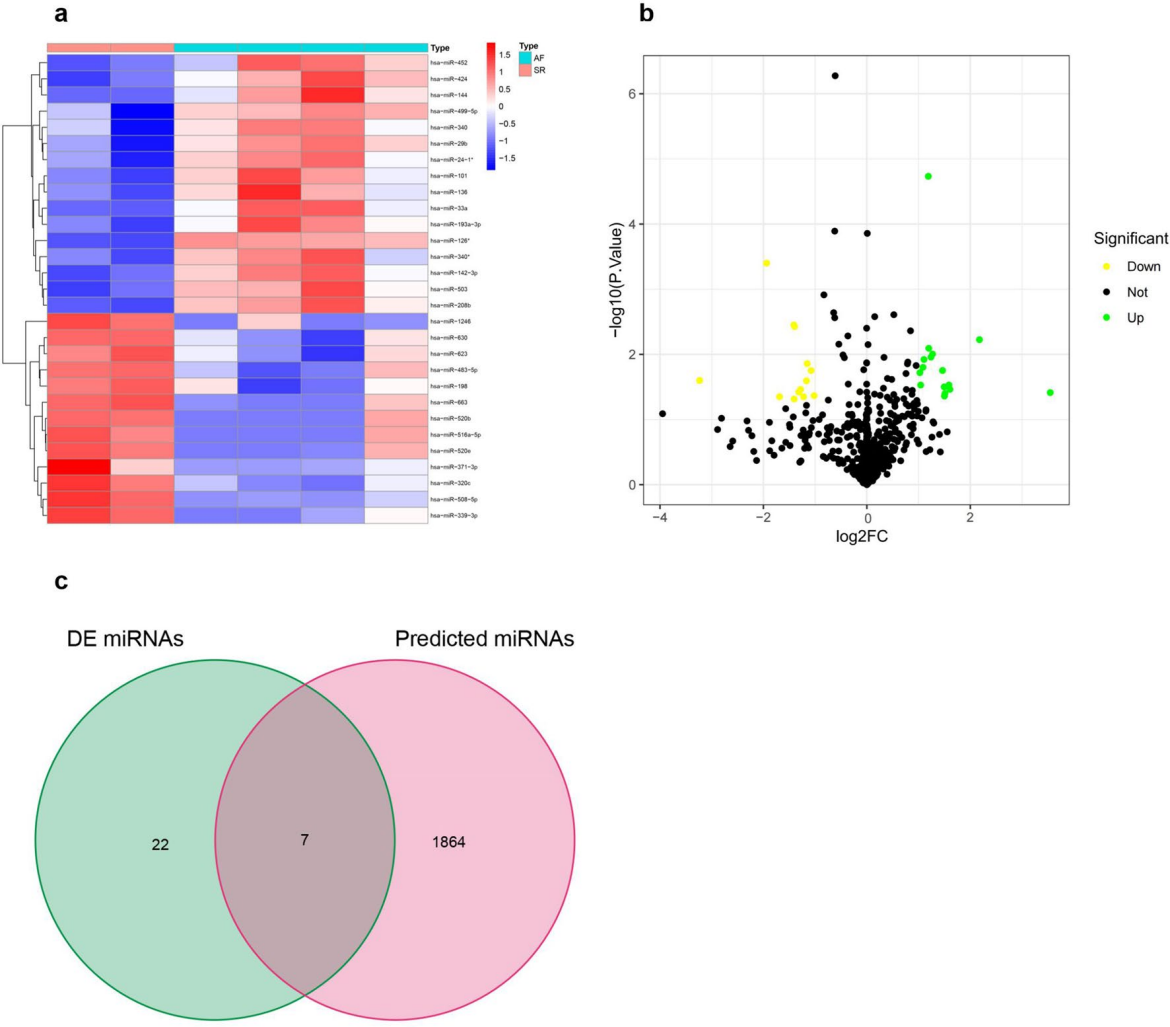
Identifying differentially expressed (DE) miRNAs with absolute |log2(fold change)|> 1 and p*-*value < 0.05 in miRNA microarray data. (**a**) The heatmap of DE miRNAs derived from the GSE70887 dataset; (**b**) volcano plot of 16 up-regulated DE miRNAs and 13 down-regulated DE miRNAs in the GSE70887 dataset; Green and yellow dots represent up and down regulated DE miRNAs, respectively; (**c**) a total of 7 overlapping miRNAs between the miRNAs predicted by DE cicrRNAs and the DE miRNAs were identified.

A total of 29 DE miRNAs (16 up-regulated and 13 down-regulated miRNAs) were screened in the GSE70887 dataset (Fig. 2a, b). According to the conditions for screening DE circRNAs in the article published by Ruan et al.^16^, a total of 120 DE circRNAs (65 up-regulated and 55 down-regulated circRNAs) were obtained.

### Construction of circRNA-miRNA network in AF

**Figure 3:**
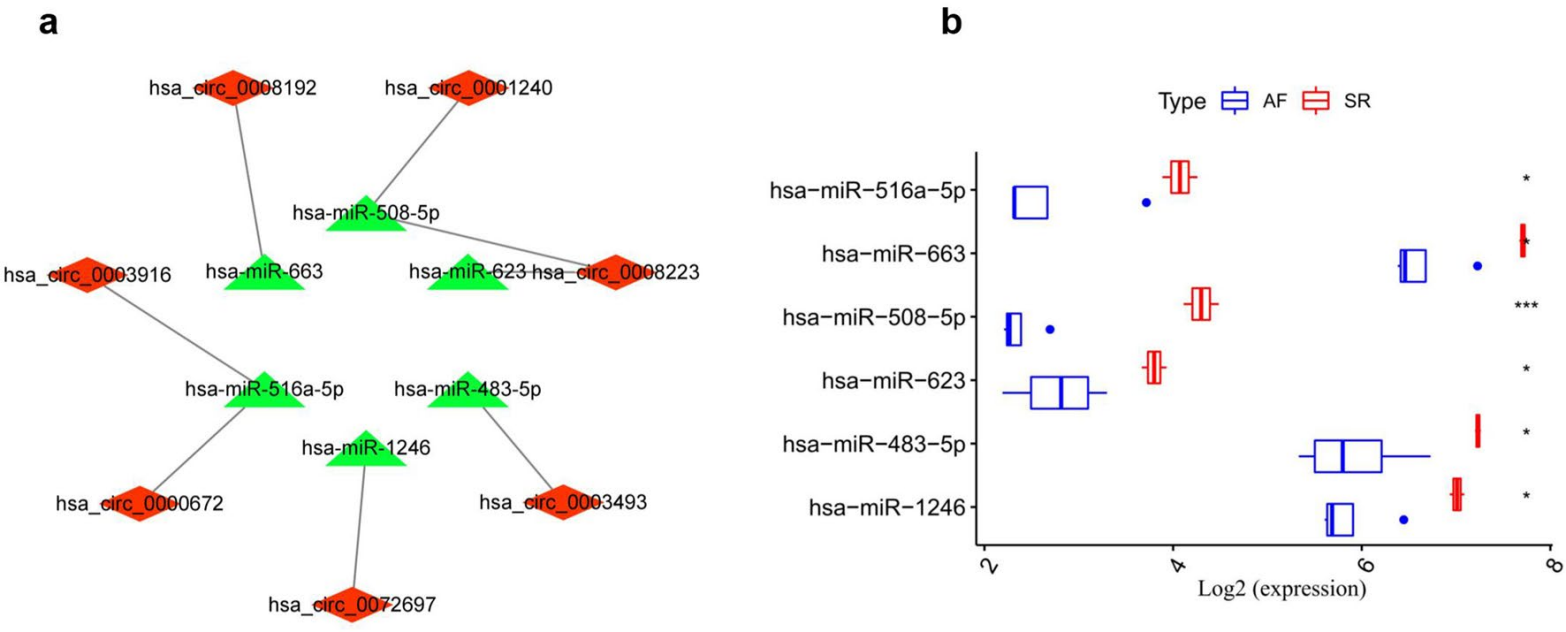
Construction of the circRNA-miRNA network in atrial fibrillation. (**a**) The circRNA-miRNA network was established by circRNA-miRNA pairs, including 7 up-regulated circRNAs and 6 down-regulated miRNAs; (**b**) boxplots of the expression levels of these 6 down-regulated miRNAs in the GSE70887 dataset. Data are shown as mean ± standard deviation, *p < 0.05, **p < 0.01, ***p < 0.001.

**Figure 4:**
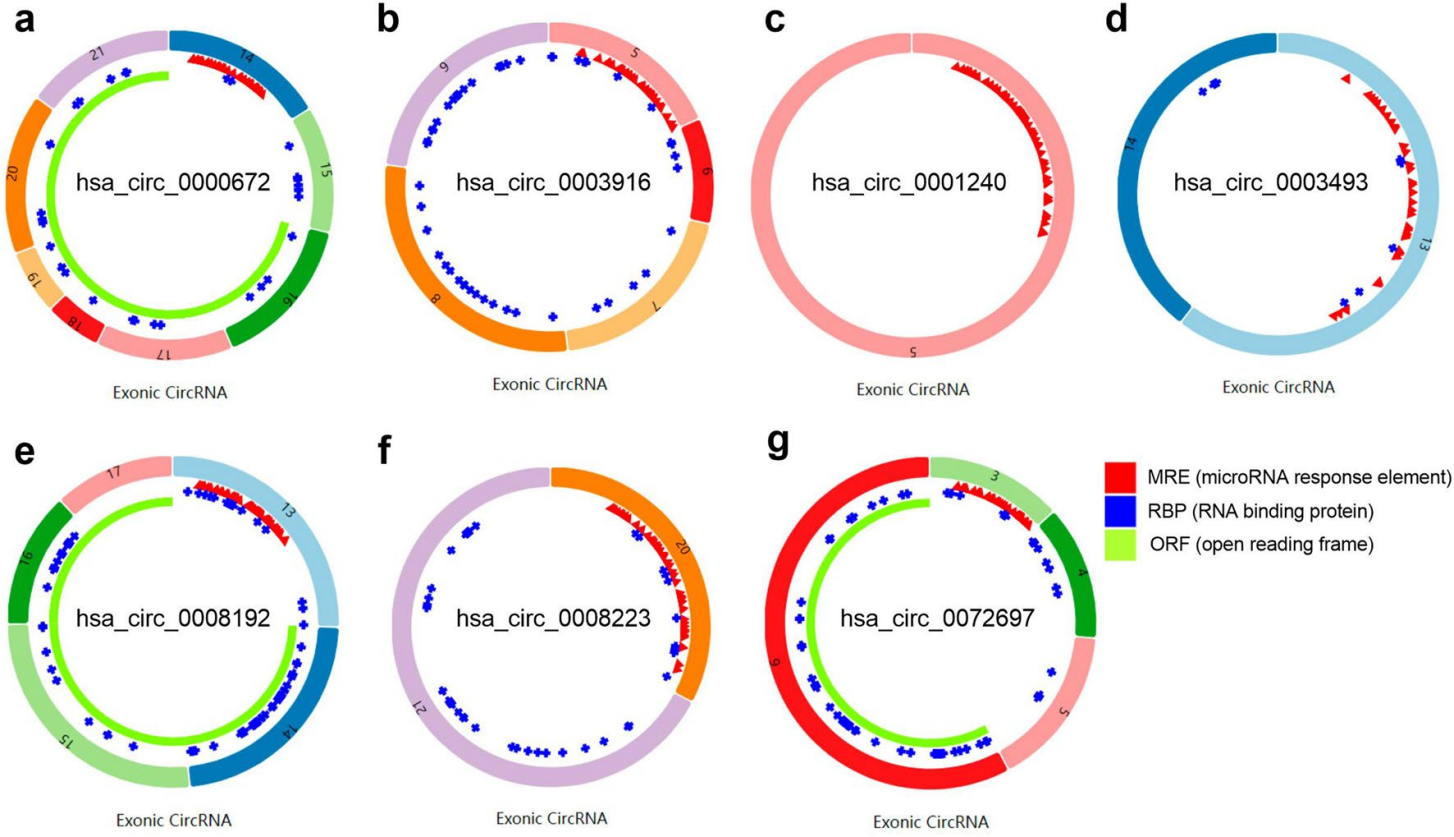
Structural patterns of the seven circRNAs in the circRNA-miRNA network (**a–g**). These structural patterns were obtained from Cancer-Specific CircRNA (CSCD). Red represents a potential position for miRNA binding, blue represents a potential position for protein binding, and yellow represents an open reading frame.

There were 48 DE circRNAs unsearched in the CSCD database among the 120 DE circRNAs. A total of 1871 targeted miRNAs were predicted using the CSCD database based on the remaining 72 DE circRNAs. After that, 7 intersecting miRNAs were obtained by intersecting DE miRNAs with target miRNAs (Fig. 2c). The circRNA-miRNA network in AF was built with 7 up-regulated circRNAs and 6 down-regulated miRNAs (Fig. 3a). In Fig. 3b, the expression levels of miRNAs in this network basing on microarray dataset were illustrated. The basic structural pattern and basic information of the 7 circRNAs in the circRNA-miRNA network were presented in Fig. 4 and Table 2, respectively.

**Table 2:** The basic information of the 7 circRNAs in the circRNA-miRNA network.

CircRNA ID	Fold change	p value	Chr	Genomic length	Strand	Gene symbol
hsa_circ_0000672	3.08	0.040	16	40,830	** + **	CLEC16A
hsa_circ_0003916	5.52	0.002	15	11,750	** + **	PIAS1
hsa_circ_0001240	6.34	0.033	22	330	**−**	NFAM1
hsa_circ_0003493	3.55	0.026	2	1754	** + **	ALS2CR8
hsa_circ_0008192	4.22	0.014	9	35,482	**−**	PTBP3
hsa_circ_0008223	2.91	0.048	16	3402	**−**	XPO6
hsa_circ_0072697	6.69	0.008	5	4774	** + **	PPWD1

Chr, chromosome.

### Functional enrichment analyses for target genes predicted by hsa-miR-516a-5p

**Figure 5:**
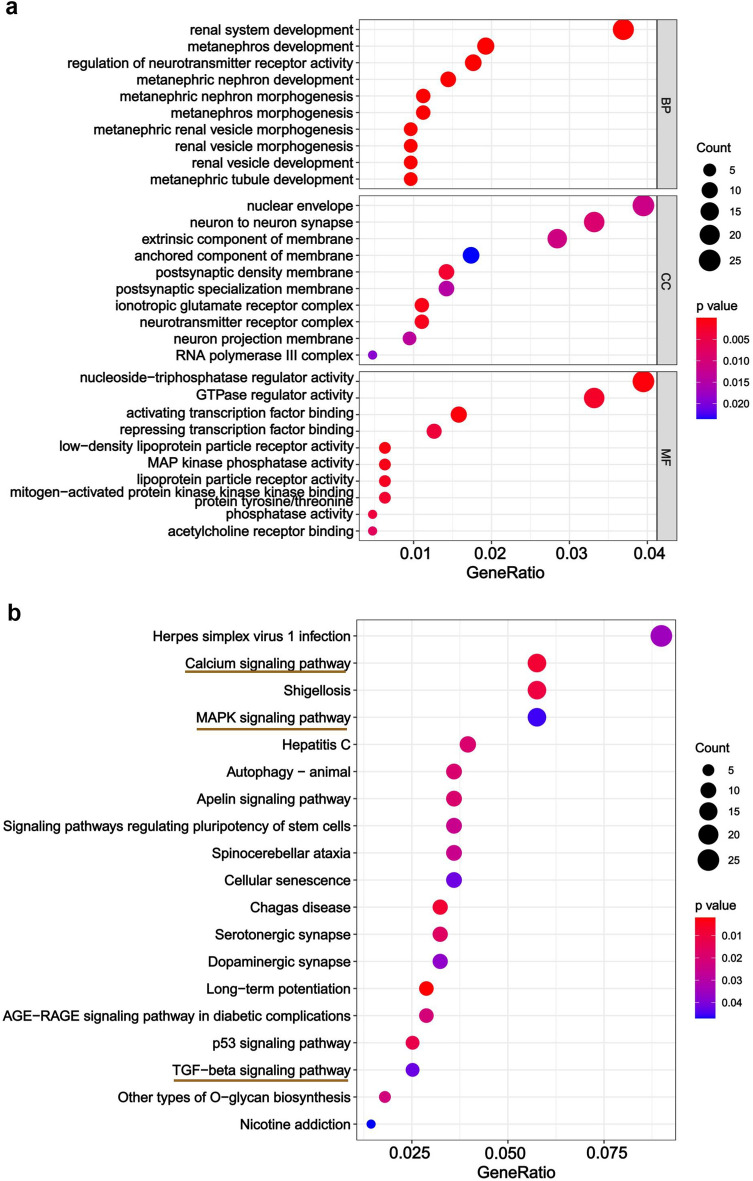
Gene ontology (GO) and Kyoto encyclopedia of genes and genomes (KEGG) pathway enrichment analysis of targeted genes predicted by miR-516a-5p. (**a**) Bubble plot of biological process (BP), cellular component (CC) and molecular function (MF); (**b**) bubble plot of KEGG pathway. Red underlined labels represent signaling pathways related to atrial fibrosis in AF.

The functional enrichment analysis of predicted target genes of each miRNA in the above constructed network revealed that only the predicted target genes of hsa-miR-516a-5p were enriched in the main signaling pathways related to atrial fibrosis in AF, and hsa-miR-516a-5p was also the miRNA that may be associated with AF in our previous study^32,33^. Consequently, the other 5 miRNAs were excluded from subsequent analysis. Using the TargetScan database, 738 potential target genes for the hsa-miR-516a-5p were predicted. The results showed that these target genes were markedly enriched in ‘renal system development’, ‘metanephros development’, and ‘regulation of neurotransmitter receptor activity’ (biological processes) (Fig. 5a); ‘nuclear envelope’, ‘neuron to neuron synapse’, and ‘extrinsic component of membrane’ (cellular components) (Fig. 5a); ‘nucleoside-triphosphatase regulator activity’, ‘GTPase regulator activity’, and ‘activating transcription factor binding’ (molecular functions) (Fig. 5a). KEGG pathway analysis revealed that the signaling pathways such as ‘Calcium signaling pathway’, ‘MAPK signaling pathway’, and ‘TGF-beta signaling pathway’, which are closely related to AF atrial fibrosis, were enriched (Fig. 5b). The 36 genes enriched in the three signaling pathways were shown in Table 3.

**Table 3:** The predicted target genes of hsa-miR-516a-5p associated with atrial fibrosis in AF according to KEGG enrichment analysis.

miRNAs	KEGG pathway	p value	Target genes
miR-516a-5p	Calcium signaling pathway	0.008	ORAI2,CACNA1C, HTR4, ITPR2, STIM2, PLCB1, GNAL, PLCD4, GRIN1, HTR2C, MYLK4, SLC25A6, TACR2, PDGFRA, CACNA1B, EDNRA
miR-516a-5p	MAPK signaling pathway	0.045	DUSP3, CACNG8, TRAF6, CACNA1C, PDGFRA, CACNA1B, RASA2, DUSP7, KRAS, DUSP1, RPS6KA2, MAPK11, MAP3K13, NFKB1, MAP3K2, DUSP8
miR-516a-5p	TGF-beta signaling pathway	0.042	SMAD6, SMAD2, ACVR2B, BMPR1B, GREM1, CREBBP, SMURF1

### Construction of PPI regulatory network and screening of hub genes

**Figure 6:**
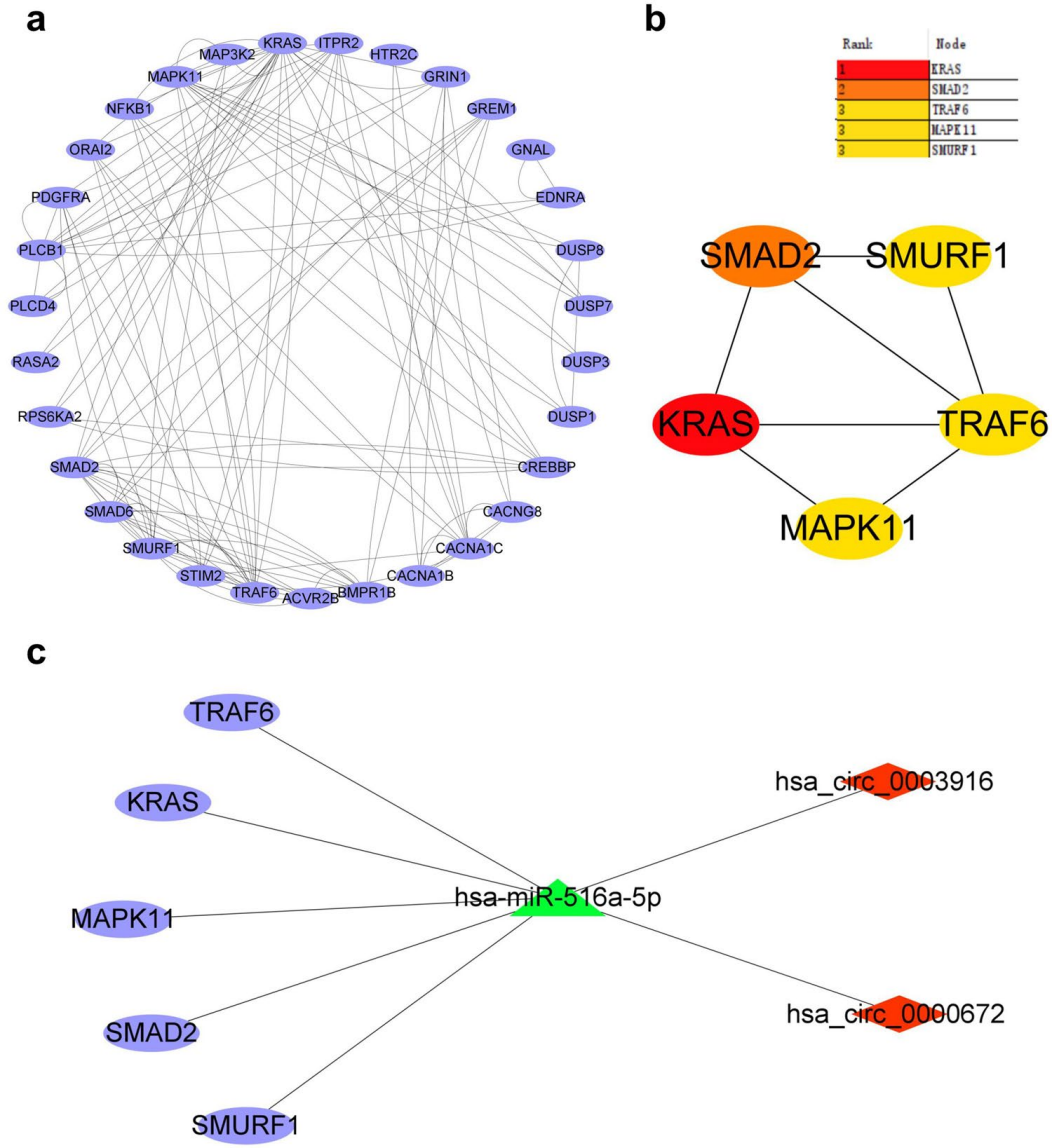
Construction of protein–protein interaction (PPI) network and circRNA-miRNA-mRNA network. (**a**) PPI network construction; (**b**) Hub genes in the PPI network were identified by using the Degree algorithm; (**c**) the circRNA-miRNA-mRNA network was constructed based on miR-516a-5p, including two up-regulated circRNAs, one down-regulated miRNA and five hub genes.

The 36 aforementioned candidate genes related to AF were used to construct PPI network by using the STRING database. Using a combined score > 0.4, the PPI network containing 31 nodes and 61 edges was formed after removing unconnected nodes (Fig. 6a). By applying the Degree algorithm to the PPI network, the hub genes that play a larger role in the PPI network and a significant module contain 5 nodes and 7 edges were identified (Fig. 6b). Then, the 5 nodes with the highest degree of connectivity were identified as the hub genes, which included V-Ki-ras2 Kirsten rat sarcoma viral oncogene homolog (KRAS), Mothers against decapentaplegic homolog 2 (SMAD2), TNF receptor-associated factor 6 (TRAF6), Mitogen-activated protein kinase 11 (MAPK11), and SMAD specific E3 ubiquitin protein ligase 1 (SMURF1), respectively (Table 4).

**Table 4:** Five hub genes in protein–protein interaction network.

Gene symbol	Degree	MCC score	Log2FC	p.adjust	Gene title
KRAS	24	23	0.77	0.0110	V-Ki-ras2 Kirsten rat sarcoma viral oncogene homolog
SMAD2	16	40	1.12	0.0002	Mothers against decapentaplegic homolog 2
TRAF6	14	16	0.86	0.0059	TNF receptor-associated factor 6
MAPK11	14	9	1.03	0.0030	Mitogen-activated protein kinase 11
SMURF1	14	33	1.36	0.0004	SMAD specific E3 ubiquitin protein ligase 1

FC, fold change; MCC, maximal clique centrality.

### Construction of circRNA-miRNA-mRNA ceRNA network related to atrial fibrosis in AF

The expression level of the above five hub genes were significantly up-regulated in AF (Table 4), by analyzing the GSE41177 dataset^34^. In addition, we found that these five hub genes were clustered in TGF-beta and MAPK signaling pathway according to the results of KEGG pathway analysis. Our above results indicated that the miRs had an opposite expression trend with the circRNAs and the hug genes in the circRNA-miRNA-mRNA ceRNA network, which is consistent with the ceRNA hypothesis^17^. Therefore, we finally constructed atrial fibrosis-related circRNA-miRNA-mRNA ceRNA network, including two up-regulated circRNAs (hsa_circ_0003916 and hsa_circ_0000672), down-regulated hsa-miR-516a-5p, and five up-regulated hub genes (KRAS, SMAD2, TRAF6, MAPK11 and SMURF1) (Fig. 6c).

Previous studies have demonstrated that SMURF1 is associated with inhibition of atrial fibrosis^35^, which is inconsistent with the predicted results of the present study. Therefore, SMURF1was excluded from this study. Up to date, there are no studies on the association of KRAS or MAPK11 with AF. The present evidences illustrate that SMAD2 and TRAF6 are involved in the process of atrial fibrosis in AF^35,36^. However, TGF-β1/SMAD2 is a known classical signaling pathway to promote atrial fibrosis^37^. Therefore, we finally focused on TRAF6, which has been rarely studied atrial fibrosis in AF, and may be a novel target for intervention of AF.

### Baseline characteristics for participants

**Figure 7:**
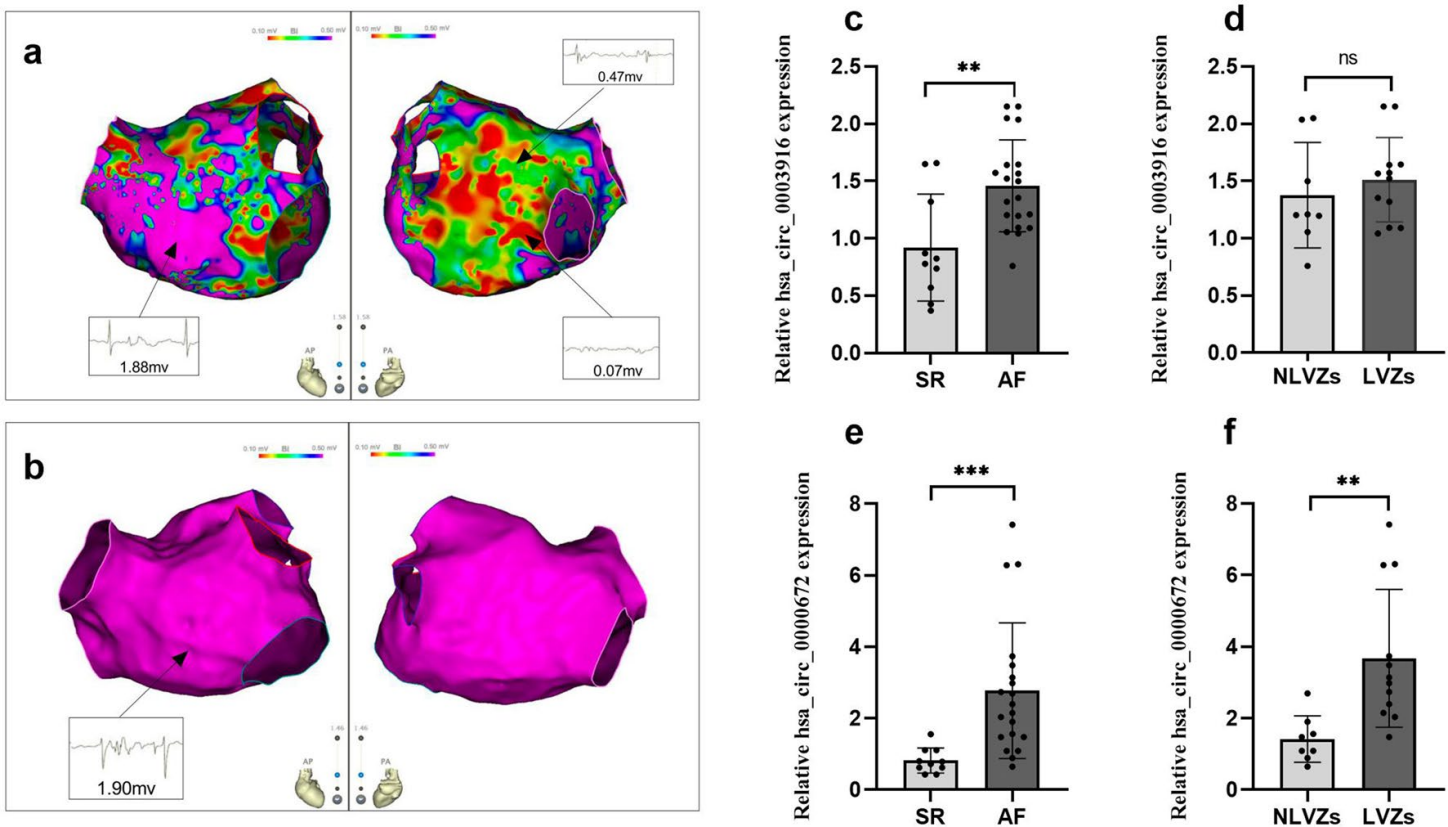
Evaluation for atrial fibrosis using LA Low-voltage mapping and two circRNAs expression in peripheral blood monocytes in patients with atrial fibrillation (AF) by qRT-PCR (**a**) A typical image of the left atrial low voltage zones (LVZs) in persistent AF, non-purple represented the LVZs (bipolar voltage < 0.5 mV), which was used to estimate atrial fibrosis, purple represented the normal substrate (bipolar voltage > 0.5 mV); (**b**) a characteristic image of the left atrial normal substrate (shown as purple) in persistent AF; (**c**) the peripheral blood monocytes hsa_circ_0003916 expression level in persistent AF and sinus rhythm (SR); (**d**) the peripheral blood monocytes hsa_circ_0003916 expression level in LVZs and non-LVZs (NLVZs) in patients with persistent AF; (**e**) a comparison of hsa_circ_0000672 expression level in peripheral blood monocytes in persistent AF and SR; (**f**) a comparison of hsa_circ_0000672 expression level in peripheral blood monocytes in LVZs and NLVZs in patients with persistent AF. Data are shown as mean ± standard deviation, **p < 0.01, ***p < 0.001.

The baseline characteristics of the participants were shown in Table 5. In PsAF group, left atrial diameter (41.3 ± 2.5 mm vs. 30.6 ± 2.9 mm, p-value < 0.001) and LVA (16.3 ± 17.5% vs. 0, p-value = 0.001) were larger than those in SR group. No significant differences on gender, age, history of diabetes, hypertension, coronary heart disease, stroke, and left ventricular ejection fraction (LVEF) were observed between the two groups. Figure 7a, b represented mapping of LA low voltage and LA normal voltage in patients with PsAF, respectively. Left atrial diameter (42.2 ± 2.7 mm vs. 40.0 ± 1.5 mm, p-value = 0.057) was not significantly increased in LVZs subgroup, and AF duration (16.8 ± 7.5 m vs. 10.6 ± 3.7 mm, p-value = 0.044) was obviously elevated in the LVZs subgroup compared to that in non-LVZs subgroup, and there were no other differences between the two subgroups.

**Table 5:** Baseline characteristics of the subjects.

Variable	PsAF group (n = 20)	SR group (n = 10)	p value
Demographics			
Age (year)	57.3 ± 11.9	49.6 ± 8.2	0.050
Male sex, n(%)	13 (65%)	5(50%)	0.429
Comorbidities			
Hypertension, n(%)	8(40%)	2(20%)	0.494
Diabetes, n(%)	1(5%)	0	1.000
Coronary heart disease, n(%)	1(5%)	0	1.000
Stroke, n(%)	0	0	1.000
Transthoracic echocardiography			
LAD (mm)	41.3 ± 2.5	30.6 ± 2.9	** < 0.001**
LVEF (%)	62.4 ± 5.0	64.3 ± 2.8	0.268
LA mapping results			
LVZs, n(%)	12(60%)	0	**0.002**
LVA (%)	16.3 ± 17.5	0	**0.001**

Significant values are in [bold].

LA, left atrial; LAD, left atrial diameter; LVA, low voltage area; LVEF, left ventricular ejection fraction; LVZs, low voltage zones; PsAF, persistent atrial fibrillation; SR, sinus rhythm.

### The expression of has_circ_0003916 and has_circ_0000672 in AF

The hsa_circ_0003916 expression in the peripheral blood monocytes was higher in the PsAF group than that in the SR group (p-value = 0.003), but not different between the LVZs subgroup and the non-LVZs subgroup (p-value = 0.473) by qRT-PCR, as shown in Fig. 7c, d. The expression of hsa_circ_0000672 was up-regulated in peripheral blood monocytes in the PsAF group, compared with that in the SR group (p-value < 0.001) by qRT-PCR, as shown in Fig. 7e. Moreover, the expression of hsa_circ_0000672 was further elevated in peripheral blood monocytes in the LVZs subgroup, compared with that in the non-LVZs subgroup by qRT-PCR, as shown in Fig. 7f (p-value = 0.002). These results demonstrated that hsa_circ_0000672 in peripheral blood monocytes may have a more close correlation with atrial fibrosis in AF than hsa_circ_0003916.

### Hsa_circ_0000672 functions as a molecular sponge of hsa-miR-516a-5p

**Figure 8:**
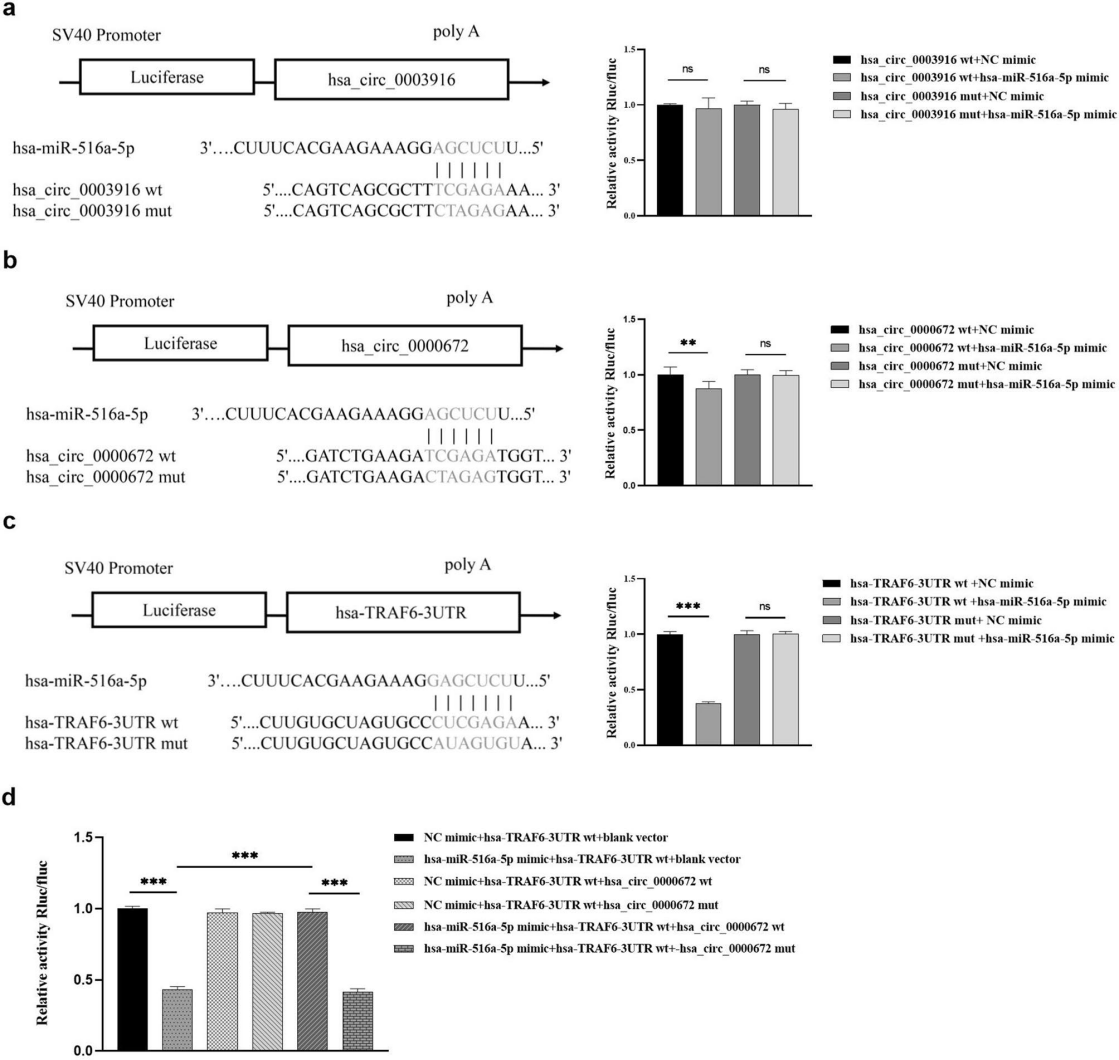
Dual luciferase assay. (**a**) The predicted binding sites of hsa-miR-516a-5p with the hsa_circ_0003916 sequence and luciferase assay of 293T cells co-transfected with hsa-miR-516a-5p mimic or negative control (NC) mimic and has_circ_0003916 wild-type (wt) or has_circ_0003916 mutation (mut) luciferase reporter; (**b**) the putative binding sites of hsa-miR-516a-5p with the hsa_circ_0000672 sequence and luciferase assay of 293T cells co-transfected with hsa-miR-516a-5p mimic or NC mimic and has_circ_0009672 wt or has_circ_0000672 mut luciferase reporter; (**c**) the binding sites of hsa-miR-516a-5p with the TRAF6 sequence and luciferase assay of 293T cells co-transfected with hsa-miR-516a-5p mimic or NC mimic and TRAF6 wt or TRAF6 mut luciferase reporter; (**d**) the luciferase activity in 293T cells that were co-transfected with TRAF6 wt luciferase reporter and hsa-miR-516a-5p mimic and blank vector was markedly decreased. However, the luciferase activity in 293T cells was rescued when transfected blank vector was replaced by hsa_circ_0000672 wt. This remedy was abolished when transfected hsa_circ_0000672 wt was replaced by hsa_circ_0000672 mut. Data are shown as mean ± standard deviation, **p < 0.01, ***p < 0.001.

Figure 8a showed the putative binding sites between hsa_circ_0003916 and hsa-miR-516a-5p. Dual-luciferase reporter assay suggested that hsa-miR-516a-5p mimics did not reduce the luciferase activity of hsa_circ_0003916 wt luciferase reporter in HEK 293T cells (Fig. 8a). The predicted binding sequence between hsa_circ_0000672 and hsa-miR-516a-5p was shown in Fig. 8b. The dual luciferase reporter assay revealed that hsa-miR-516a-5p mimics reduced the luciferase activity of the hsa_circ_0000672 wt luciferase reporter, but cannot decrease the luciferase activity of hsa_circ_0000672 mut luciferase reporter (Fig. 8b). These results confirmed that hsa_circ_0000672 directly bound hsa-miR-516a-5p, and decreased the level of free hsa-miR-516a-5p. However, hsa_circ_0003916 had no similar effect on hsa-miR-516a-5p.

### TRAF6 is a direct target gene of hsa-miR-516a-5p

The predicted binding sites of hsa-miR-516a-5p with TRAF6 were shown in Fig. 8c. In HEK 293T cells, hsa-miR-516a-5p mimic attenuated the luciferase activity of TRAF6-3′-UTR wt luciferase reporter, but cannot suppress the luciferase activity of TRAF6-3′-UTR mut luciferase reporter by the dual-luciferase reporter assay (Fig. 8c). The results revealed that TRAF6 is the direct target gene of hsa-miR-516a-5p.

### Hsa_circ_0000672 regulates the expression level of TRAF6 by competitively binding hsa-miR-516a-5p with TRAF6 in HEK 293T cells

The luciferase activity was markedly decreased in HEK 293T cells that were co-transfected with TRAF6-3’-UTR wt luciferase reporter, hsa-miR-516a-5p mimic, and blank vector. However, the luciferase activity was rescued in HEK 293T cells, when transfected blank vector was replaced by hsa_circ_0000672 wt plasmid. This remedy was abolished when transfected hsa_circ_0000672 wt plasmid was replaced by hsa_circ_0000672 mut plasmid (Fig. 8d). These results suggested that there was a competitive relationship between hsa_circ_0000672 and TRAF6 for hsa-miR-516a-5p binding.

## Discussion

Atrial structural remodeling characterized by atrial fibrosis is considered to be the main pathological mechanism for the initiation and maintenance of AF, and it is difficult to reverse^3,4^. Moreover, atrial fibrosis is an independent risk factor for AF recurrence after radiofrequency ablation^6^. However, the molecular mechanism of atrial fibrosis is still not fully elucidated. Thus, a further understanding on the molecular mechanism of atrial fibrosis is particularly important to find the targets for early intervention of atrial structural remodeling.

In this study, we first constructed the circRNA-miRNA network associated with AF, which included 7 up-regulated circRNAs and 6 down-regulated miRNAs, by analyzing the DE circRNAs and DE miRNAs. Analysis of these 6 miRNAs in this network, a study has reported that hsa-miR-663 can prevent monocrotaline-induced pulmonary arterial hypertension by targeting TGF-β1/smad2/3 signaling^38^. We speculate that low-expressed hsa-miR-663 may be involved in the development of atrial fibrosis through fibrosis-related TGF-β1/smad2/3 signaling pathway^37^. It has also been reported that the hsa-miR-508-5p-SMOC2 regulatory axis may be involved in the inflammatory damage in AF^39^. No studies have reported that hsa-miR-623, hsa-miR-483-5p or hsa-miR-1246 is associated with AF. In the following circRNA-miRNA-mRNA ceRNA network construction, aforementioned 5 miRNAs were excluded since the target genes predicted by these 5 miRNAs were not enriched in the major pathways related to atrial fibrosis in AF.

For hsa-miR-516a-5p, we found that it was once predicted to be in the circRNA-miRNA-mRNA ceRNA network related to AF that we built previously^32,33^, which indicated that hsa-miR-516a-5p may be potentially involved in the progression of AF. At present, miR-516a-5p has been found to act as a tumor suppressor in non-small cell lung cancer^40^, hepatocellular carcinoma^41^, and bladder cancer^42^, but there are few studies on its role in AF. The predicted target genes of miR-516a-5p were enriched in signaling pathways correlated with atrial fibrosis in AF, such as the “Calcium signaling pathway”, “MAPK signaling pathway”, and “TGF-beta signaling pathway”, according to KEGG pathway analysis. These results helped us better understand the functions of this miRNA associated with atrial fibrosis in AF. A large amount of evidences have indicated that intracellular Ca^2+^ dysregulation plays a key role in the initiation and maintenance of AF, and also participates in the process of atrial fibrosis^43,44^. There have been growing evidences that MAPK signaling pathway contributes to the pathogenesis of atrial fibrosis in recent years^45,46^. TGF-β1 has been shown to be associated with the occurrence and progression of AF through a well-known pro-fibrotic mechanism^47^, and a recent study has shown that miR-181b mediates TGF-β-induced endothelial-mesenchymal transition involved in AF by targeting semaphorin 3A^48^. These three signaling pathways are implicated in the progression of atrial fibrosis in AF. Therefore, we speculate that miR-516a-5p may be involved in atrial fibrosis, but its specific mechanism needs to be further explored.

Next, we constructed a PPI network using these 36 genes enriched in the three signaling pathways, and identified five hub genes (KRAS, SMAD2, TRAF6, MAPK11, and SMURF1), which were enriched on TGF-β and MAPK signaling pathways. Subsequently, we used the GSE41177 dataset to confirm that the expression level of these five hub genes in AF were significantly up-regulated. Finally, the circRNA-miRNA-mRNA ceRNA network associated with atrial fibrosis in AF was constructed, which included two up-regulated hsa_circ_0003916 and hsa_circ_0000672, down-regulated hsa-miR-516a-5p, and five up-regulated hub genes.

TRAF6 is a member of a family of six TRAF molecules found in mammals and its biological effects are mostly achieved through signal transduction pathways^49^. A study by Zhang et al.^50^ showed that TRAF6 expression was significantly increased in chronic AF patients, and TRAF6 was involved in atrial remodeling. There is an evidence that TRAF6/TGF β-associated kinase 1 (TAK1) plays a critical role in the TGF-β1/non-Smad signaling pathway correlated with atrial fibrosis^49^. Meanwhile, TRAF6/TAK1 pathway is implicated in angiotensin II (AngII)-induced atrial fibrosis. Furthermore, the proliferation of atrial fibroblasts caused by AngII is attenuated by TRAF6 siRNA^36^.

Our results showed that has_ circ_0003916 and has_ circ_0000672 were elevated in patients with AF. Recently, a study has shown that hsa_circ_0000672 is involved in SiO2-induced pulmonary fibrosis^51^, but there is no reporter on circ_0003916 related to fibrosis. In our study, LA fibrosis was evaluated by LA low voltage using high-density bipolar LA voltage mapping^7^. Our results showed that the hsa_circ_0003916 expression in the peripheral blood monocytes was not different between the LVZs subgroup and the non-LVZs subgroup, but the expression of hsa_circ_0000672 was elevated in peripheral blood monocytes in the LVZs subgroup, compared with that in the non-LVZs subgroup. These results further suggest that hsa_circ_0000672 may have a more close correlation with atrial fibrosis in AF than hsa_circ_0003916. Therefore, we speculated that hsa_circ_0000672 may activate TRAF6/TAK1 signaling pathway to promote atrial fibrosis by competitively binding hsa-miR-516a-5p with TRAF6. To verify this hypothesis, we performed 3 dual luciferase assays. First, hsa_circ_0000672 and TRAF6 were certified as direct target gene of hsa-miR-516a-5p, which is a basis of hsa_circ_0000672 as a ceRNA of TRAF6. To further clarify that hsa_circ_0000672 regulates expression of TRAF6 as a ceRNA by sponging hsa-miR-516a-5p, a dual luciferase assay was performed in HEK 293T cells triple transfected with fluorescent luciferase reporter plasmids containing TRAF6-3′-UTR wt or TRAF6-3’-UTR mut, and plasmids containing has_circ_0000672 wt, hsa_circ_0000672 mut, or blank vector, and hsa-miR-516a-5p mimic or negative control mimic. The results uncovered that the luciferase activity was markedly decreased in HEK 293T cells that were co-transfected with TRAF6-3′-UTR wt luciferase reporter, hsa-miR-516a-5p mimic and blank vector. However, the luciferase activity was rescued when blank vector was replaced by hsa_circ_0000672 wt plasmid. This remedy was abolished when hsa_circ_0000672 wt plasmid was replaced by hsa_circ_0000672 mut plasmid. These evidences support our hypothesis in vitro. In the other hand, hsa_circ_0003916 did not function as ceRNA for sponging miR-516a-5p in our study. Therefore, the specific mechanism needs to be further clarified, although hsa_circ_0003916 might be associated with the process of AF.

In this study, there were some limitations. First, the sample sizes of the validation were relatively small. In order to establish the role of hsa_circ_0000672 as a biomarker in predicting atrial fibrosis in AF, we need a large sample multicenter cohort study in further. Second, the LVZs may not completely represent degree of actual atrial fibrosis because it has not been confirmed histologically, but it is not possible to use histological evaluation because of invasion. It may further increase the reliability for evaluation of atrial fibrosis to use LGE-MRI simultaneously. However, the cost of LGE-MRI is expensive, which will increase economic burden for patients. Moreover, recent evidences have revealed that LVZs have a close correlation with the fibrosis region quantified by LGE-MRI^52^. Therefore, we think that LA Low-voltage mapping may be the most feasible method to evaluate atrial fibrosis in the present study. Third, in order to further search for ceRNA networks related to atrial fibrosis in AF, we did not extensively analyzed circRNA-miRNA network pertaining to AF and their target genes. In addition, in the circRNA-miRNA-mRNA ceRNA network that we built by bioinformatics analysis, we only identified that hsa_circ_0000672 indirectly regulated TRAF6 as a ceRNA by binding to miR-516a-5p in vitro. However, whether hsa_circ_0000672 has a similar mechanism to regulate other hub genes including KRAS, SMAD2, and MAPK11 in this ceRNA network, has not been verified in the present study. We plan to finish these works in our further research. Lastly, it is only verified that hsa_circ_0000672 indirectly regulates the expression level of TRAF6 by competitively binding hsa-miR-516a-5p with TRAF6 in vitro in our study. We need to testify this mechanism in vivo in further.

## Conclusions

In conclusion, the present study found the increased expression of hsa_circ_0000672 in the peripheral blood monocytes of patients with PsAF, especially in PsAF patients with LVZs. hsa_circ_0000672 may positively regulated the expression of TRAF6 to promote atrial fibrosis via acting as a ceRNA by sponging hsa-miR-516a-5p in vitro. hsa_circ_0000672 may be a peripheral blood biomarker for atrial fibrosis, and hsa_circ_0000672/hsa-miR-516a-5p/TRAF6 axis may be a novel intervention target for atrial fibrosis in AF.

## Data Availability

The original contributions to this study has been included in the article, and further inquiries can be directed to the corresponding author.
